# Biosimilars: Key regulatory considerations and similarity assessment tools

**DOI:** 10.1002/bit.26438

**Published:** 2017-09-19

**Authors:** Carol F. Kirchhoff, Xiao‐Zhuo Michelle Wang, Hugh D. Conlon, Scott Anderson, Anne M. Ryan, Arindam Bose

**Affiliations:** ^1^ Pfizer Inc Global Technology Services Biotechnology and Aseptic Sciences Group Chesterfield Missouri; ^2^ Pfizer Inc BioTherapeutics Pharmaceutical Sciences Pearl River New York; ^3^ Pfizer Inc Analytical Research and Development Andover Massachusetts; ^4^ Pfizer Inc Worldwide Regulatory San Diego California; ^5^ Pfizer Inc Drug Safety Research and Development Groton Connecticut; ^6^ Pfizer Inc BioTherapeutics Pharmaceutical Sciences Groton Connecticut

**Keywords:** analytical characterization, biologic, biosimilar, development, manufacturing, regulatory requirements

## Abstract

A biosimilar drug is defined in the US Food and Drug Administration (FDA) guidance document as a biopharmaceutical that is highly similar to an already licensed biologic product (referred to as the reference product) notwithstanding minor differences in clinically inactive components and for which there are no clinically meaningful differences in purity, potency, and safety between the two products. The development of biosimilars is a challenging, multistep process. Typically, the assessment of similarity involves comprehensive structural and functional characterization throughout the development of the biosimilar in an iterative manner and, if required by the local regulatory authority, an in vivo nonclinical evaluation, all conducted with direct comparison to the reference product. In addition, comparative clinical pharmacology studies are conducted with the reference product. The approval of biosimilars is highly regulated although varied across the globe in terms of nomenclature and the precise criteria for demonstrating similarity. Despite varied regulatory requirements, differences between the proposed biosimilar and the reference product must be supported by strong scientific evidence that these differences are not clinically meaningful. This review discusses the challenges faced by pharmaceutical companies in the development of biosimilars.

## 1 INTRODUCTION

Biologic products (also termed “biopharmaceuticals”) are most often produced by living systems, and are typically manufactured using cell culture of genetically engineered animal, bacterial, or plant systems. In contrast, small‐molecule drugs are chemically synthesized and, therefore, involves a more straightforward process to produce an identical (generic) product following patent expiration of the approved product (Nowicki, 2007).

Biologic products are an important treatment option for a wide array of conditions and diseases, primarily cancer, rheumatoid arthritis, and inflammatory bowel disease (Walsh, 2010), either to supplement small‐molecule drugs or as stand‐alone medications. Patents for some currently licensed biologic products have already expired or will expire in the coming years. Moreover, 7 of the top 10 pharmaceutical products sales in 2016 were biologic drugs (EvaluatePharma, 2017). As such, the concept of developing biologic products that are “biosimilar” (highly similar) to the approved biologic products has aroused great interest worldwide, across government bodies as well as in scientific and medical communities (Li et al., 2015). Biosimilars may offer increased treatment options for patients and physicians, and may optimize efficiencies across healthcare systems. Therefore, biosimilars have the potential to provide lower cost alternatives and offer greater access to biologics, and thereby allow increased use of biologic therapies (Baer, Maini, & Jacobs, 2014).

In broad terms, a biosimilar is highly similar to a reference product in terms of structure and function (WHO, 2016). The development of biosimilars is associated with numerous challenges, including the proprietary nature of the production processes of the reference product (the approved product) and the complexity of biologic molecules (Bandyopadhyay, 2013). By definition, and in contrast with small‐molecule generic products, it is impossible to manufacture identical copies of biologic products (WHO, 2016). However, information from European Public Assessment Reports, US Summary Basis of Approval, published literature, and requests via the Freedom of Information Act (EMA, 2017; FDA, 2015a) can be harnessed to obtain methodology and results from key studies pertaining to the reference product. Together with the biosimilar developer's knowledge of analytical characterization and manufacturing, these data act as the basis for a strategy for biosimilar development.

Additional information on the reference product is acquired through extensive structural and functional characterization encompassing many product quality attributes, including primary sequence, higher order protein structure, post‐translational modifications, protein aggregation and product‐related impurities, and biological activities (Tsuruta, Lopes dos Santos, & Moro, 2015). Through a process of reverse engineering, a manufacturing process is developed that results in a biologic product that is highly similar to the reference product (Tsuruta et al., 2015). Because the development of a biosimilar likely begins several years after the development of the reference product, the relevant technologies may have evolved in the intervening years. The regulatory expectation is that the biosimilar manufacturer applies contemporary technologies in their product development, and must also adhere to current industry standards and regulatory expectations, which may also have evolved from the time that the reference product was developed and approved.

The approval of biosimilars is a highly regulated and detailed process. The European Medicines Agency (EMA) and the United States (US) Food and Drug Administration (FDA) guidance documents stipulate that a biosimilar manufacturer must perform a series of extensive similarity assessments in order to demonstrate biosimilarity to the reference product, and to ultimately gain regulatory approval or licensure (EMA, 2015; FDA, 2015b) (Table 1). The World Health Organization (WHO) has also published general guiding principles for the development of biosimilars, with the aim of providing a coherent approach for national regulatory guidelines (WHO, 2016).

**Table 1:** Global variations in the regulatory approval processes of biosimilars

Regulatory body	Definition and key criteria
EMA (EMA, 2015)	A biological medicinal product that contains a version of the active substance of an already authorized product (reference medicinal product) in the EEA
FDA (FDA, 2015b)	A biological product that is highly similar to a US‐licensed reference product notwithstanding minor differences in clinically inactive components, and for which there are no clinically meaningful differences between the biological product and the reference product in terms of safety, purity, and potency of the product
WHO (WHO, 2016)	A biotherapeutic product that is similar in terms of quality, safety, and efficacy to an already licensed reference product

EEA, European Economic Area; EMA, European Medicines Agency; FDA, US Food and Drug Administration; WHO, World Health Organization.

Globally, regulatory expectations for the development and approval of biosimilars are not completely harmonized. Regional‐ and country‐specific biosimilar pathway legislation and guidance are at different stages of development and implementation. As a result, there is no global harmonization on certain aspects of biosimilar development, including the selection of the reference product, nomenclature, and the design of analytical, non‐clinical, or clinical comparative studies. Indeed, global agreement on the regulatory requirements will optimize the development and manufacturing of biosimilars worldwide.

The purpose of this review is to discuss some of the challenges of biosimilar development.

## 2 REGULATORY REQUIREMENTS FOR BIOSIMILARS: RIGOROUS, COMPREHENSIVE, AND EVOLVING

Demonstrating biosimilarity requires rigorous evaluation of the proposed biosimilar including side‐by‐side comparison with the reference product. During the development of the reference product, the developer must conduct extensive preclinical studies and large clinical trials in all indications for which approval will be sought. However, for a biosimilar developer, the comparative analytical characterization and the demonstrated similarity between a proposed biosimilar and the reference product reduces the requirement for large clinical trials in all the indications approved for the reference product (WHO, 2016).

The biosimilars approval pathway was pioneered in the European Union (EU), which has established regulatory architecture, with 11 product classes (under 31 different trade names) currently authorized by the EMA (EMA, 2017). The FDA has also developed extensive guidance on the regulatory requirements for the evaluation of similarity and granted approval of five biosimilars to date (Table 2).

**Table 2:** FDA approval of biosimilars in the United States

Manufacturer	Originator (reference) product	Biosimilar	Approval year/supporting reference
Sandoz	Fligrastim	Zarxio®	2015 (Sandoz Inc, 2015)
Amgen	Adalimumab	Amjevita®	2016 (FDA, 2016a)
Sandoz	Etanercept	Erelzi®	2016 (FDA, 2016b)
Celltrion	Infliximab	Inflectra®	2016 (Pulse, 2016)
Samsung Bioepis	Infliximab	Renflexis®	2017 (Samsung Bioepsis, 2017)

FDA, US Food and Drug Administration.

**Figure 1:**
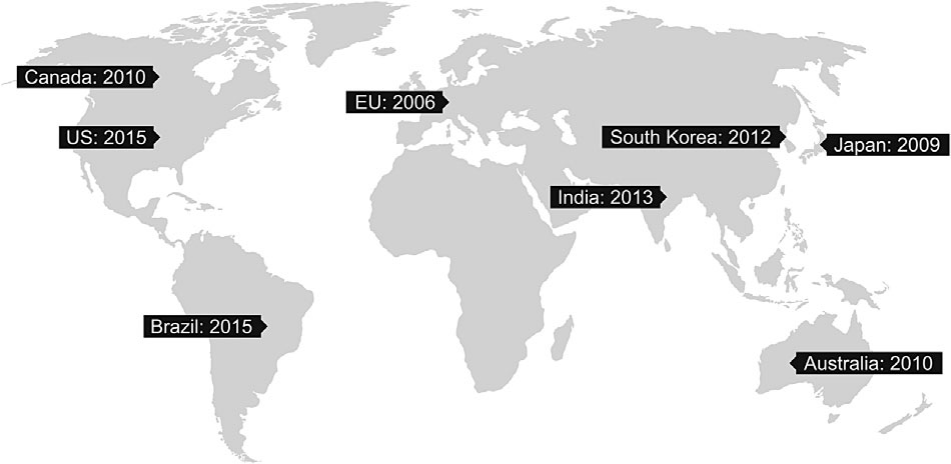
Evolution of the biosimilars regulatory landscape across the globe (Krishnan et al., 2015; WHO, 2016)

The ultimate goal of the regulatory bodies is to ensure that biosimilars meet high standards of quality, safety, and efficacy, and are highly similar to the reference product. However, although there are many regulatory guidance documents, there is no global consensus on the regulatory approval pathway for biosimilars. Many countries, besides the United States and EU, are currently authoring guidance documents for biosimilars (Casey, 2016). Several, including Canada, Brazil, South Africa, Japan, and Korea have used the principles for establishing biosimilarity outlined in the WHO guidance documents as a platform for authoring their national guidelines (Krishnan, Mody, & Malhotra, 2015; WHO, 2016) (Figure 1).

### 2.1 Evidence requirements for the approval of biologics and biosimilars: A different way of thinking

**Figure 2:**
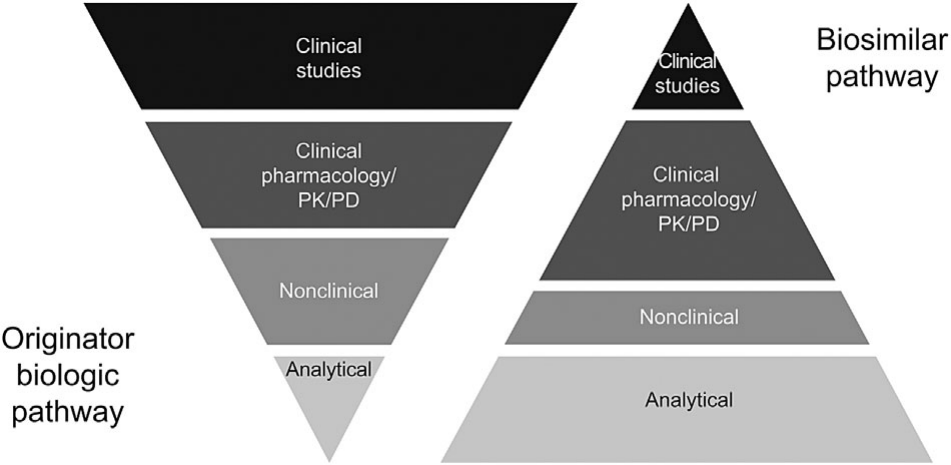
Biologics and biosimilars: an alternative approach to development. Adapted from Kozlowski (2012)

The development pathway of an originator biologic requires extensive clinical evaluations, with the ultimate aim of establishing superiority (vs. placebo or comparator agents) in terms of efficacy and an adequate safety profile. In contrast, the pathway for biosimilar development is to demonstrate similarity to the reference product with respect to quality, safety, and efficacy using a stepwise approach that includes analytical, nonclinical, and clinical studies, rather than establish de novo safety and efficacy (FDA, 2015b) (Figure 2).

Based on this model, biosimilarity is evaluated using a scientifically tailored approach, with approval based on the “totality of the evidence,” including analytical, (structural and functional), animal toxicity, pharmacokinetic (PK), pharmacodynamic (PD), immunogenicity, and clinical safety and effectiveness (FDA, 2015b). Individual regulatory agencies across the globe determine biosimilarity by assessing all of the available data provided by the biosimilar developer. As such, a biosimilar may be deemed similar to a reference product even if there are minor analytical differences between the two, provided that sufficient scientific data and appropriate justification are supplied to show that these differences are not clinically meaningful (FDA, 2015b). Throughout the development of biosimilars, the nature and potential impact of residual uncertainty are evaluated and addressed at each stage and, in certain cases, may warrant the need for additional studies (FDA, 2015b).

## 3 SELECTION OF REFERENCE PRODUCTS

There is currently no global consensus regarding the source of reference products, although WHO guidelines have outlined fundamental principles and key considerations in the selection of reference products for the development of biosimilars (WHO, 2016). WHO guidance documents state that a reference product is “the comparator used for head‐to‐head comparability studies with the similar biotherapeutic product in order to demonstrate similarity in terms of quality, safety, and efficacy” (WHO, 2016). Further, WHO guidelines stipulate that only a reference product that was licensed on the basis of a complete registration dossier can serve as a reference product (i.e., an approved biosimilar cannot serve as the reference product in the development of another biosimilar). The EMA guidance document states that a single reference product, defined on the basis of its marketing authorization in the European Economic Area (EEA), should be used as the reference product throughout the similarity exercise for quality, safety, and efficacy studies during the development of a proposed biosimilar to facilitate a coherent approach throughout the process (EMA, 2015). With the overarching goal of encouraging the global development of biosimilars and to circumvent repetition of clinical studies, there is a certain degree of flexibility in selecting a reference product. In light of this, the EMA has recently indicated a degree of acceptance of reference products authorized outside the EEA, provided that it is representative of the reference product authorized within the EEA (EMA, 2015).

The FDA accepts the use of a reference product that is not authorized in the United States, provided that comparability between the US‐ and non‐US‐licensed reference products has been adequately demonstrated (FDA, 2015b). Indeed, bridging the selection of reference products licensed in the United States to the EU‐approved reference product will likely facilitate the ongoing harmonization of biosimilars development. Guidelines in Canada and Republic of Korea state that the reference product should be authorized within a well‐established regulatory framework (Health Canada, 2016; National Institute of Food and Drug Safety Evaluation in Korea, 2015). Indeed, the use of a globally accepted reference product would allow manufacturers to reduce the number of human subjects and possibly clinical trials required for global approval of potential biosimilars (GaBI, 2014).

## 4 MANUFACTURING OF BIOSIMILARS: A CHALLENGING PROCESS

Due to their size and complexity, as well as differences in host cell lines and biological expression systems, the manufacture of biologics, including biosimilars, is challenging (Schiestl, Zabransky, & Sorgel, 2017). Control of biological expression systems is complex, and even small changes in bioreactor parameters may influence the clinical performance of a potential biosimilar. Factors such as pH, temperature, oxygen, light, forces experienced during cell culture, purification, formulation, and storage can also influence the quality of potential biosimilars throughout the manufacturing process (Mellstedt & Ludwig, 2008). In addition, the structure, biological activity and intrinsic stability can be influenced by events that occur throughout the manufacture of biosimilars. As such, caution must be exercised throughout the entire manufacturing process to avoid structural changes. Moreover, developers maintain strict control over the quality of incoming raw materials and extensively characterize their manufacturing processes to maintain batch‐to‐batch variability within an acceptable range (Kresse, 2009; Mellstedt & Ludwig, 2008).

The proprietary nature of the manufacturing process of the reference product is a key challenge in the development and production of biosimilars. Biosimilars may be developed one to two decades after the initial approval of the reference product; therefore, where literature descriptions of the original process may exist, they may be of little value. Owing to advances in science and manufacturing technology in the intervening years, requirements may have evolved since the time of licensure of the reference product. Indeed, both the EMA and FDA allow for advancements in formulation science to be incorporated in the biosimilar presentation (i.e., the formulation excipients in the biosimilar may differ from those of the reference product), and assessments are undertaken to elucidate any relevant effects of the revised formulation on the stability, physiochemical, and functional characteristics of biosimilars (EMA, 2015; FDA, 2015b).

Biosimilar developers use the same manufacturing principles, basic processes, and current good manufacturing practices (cGMP) as those of the originator biologic (FDA, 2015b). The manufacture of biosimilars is a multistep process, beginning with the selection of an appropriate host cell line and transfecting the host with DNA that encodes the protein sequence of the reference product. Routine production of biologic products and biosimilars include fermentation, purification, formulation, fill, and finish, followed by analytical testing of the product. For biosimilars, determining the correct protein sequence of the reference product, and thus correctly encoding the DNA to be transfected into the host cell is a challenge because, although literature and patent information contain protein sequence details, this information can often be misleading or incomplete (Nowicki, 2007). Therefore, the biosimilar developer must confirm the amino acid sequence of the reference product prior to constructing the DNA sequence. The optimum host cell line for production is subsequently identified, based on product quality, cell growth, and protein expression characteristics. After transfecting a host cell, the specific clone is chosen principally based on the desired/critical product attributes of the protein produced. This is an iterative process to identify not only an appropriate clone, but also the production conditions that will deliver target product quality attributes similar to those of the reference product (Lee, Litten, & Grampp, 2012).

The purification process of biologic products and biosimilars involves chromatographic and filtration steps. This too is based on the biosimilar developer's manufacturing knowledge and is an iterative process to select the purification process that will deliver target product quality attributes similar to those of the reference product and meet current product safety standards and expectations.

Differences between the proposed biosimilar and the reference product may arise due to differences in host cell line, growth media, culture conditions such as temperature, pH, and agitation rate, as well as differences in the purification process (Mellstedt & Ludwig, 2008). However, the biosimilar developer must demonstrate similarity of target product quality attributes with the highest scrutiny on those that impact the mechanism of action of the biotherapeutic. The characterization of multiple lots of the reference product at the outset and, thereafter at regular intervals, enables the design of an appropriate and robust manufacturing process, which will consistently produce a highly similar product. Manufacturing consistency ensures a product that meets approved specifications over the life cycle of a product (FDA, 2015b).

During development, manufacturing processes are designed, developed, and understood through the science‐ and risk‐based approaches of quality by design. Quality by design is defined as a “systematic approach to development that begins with predefined objectives and emphasizes product and process understanding and process control, based on sound science and quality risk management (FDA, 2012).”

It is particularly important that the product quality attributes of the reference product, which are pertinent to the intended clinical profile (known as critical quality attributes [CQAs]), are within an appropriate range, limit, or distribution for the proposed biosimilar. CQAs are product‐specific. The primary amino acid sequence must be identical to the reference product as the sequence is critical in determining the structure and biological activity of a biologic product. Other CQAs likely include aggregate levels, bioactivity, charge heterogeneity, and the glycosylation profile, particularly for monoclonal antibodies (mAbs), which may be part of the mechanism of action of the biologic (ICH, 2009; Tsuruta et al., 2015). While biosimilars guidelines allow for the use of different cell lines for the biosimilar than that used by the originator, particular attention should be paid to quality attributes that may be impacted by the cell line chosen, such as glycosylation (EMA, 2015; FDA, 2015b).

Once the cell culture conditions and purification process are optimized, the production process is scaled up to the proposed commercial scale and refined to maximize product yield, while maintaining product quality attribute ranges of the proposed biosimilar. The biosimilar manufacturer must consider potential lot‐to‐lot variability in quality attributes and carefully examine changes across and within multiple production runs (Tsuruta et al., 2015). The stability of the proposed biosimilar versus the reference product should also be assessed under various stress conditions, such as light and accelerated temperature conditions (EMA, 2015).

Manufacturing process control is conducted to deliver the necessary degree of consistency (Ramanan & Grampp, 2014). Overall product control testing includes evaluations of raw materials, cell‐lines, in‐process sampling and testing throughout the manufacturing process, analytical release, and stability testing over the storage conditions and time period. The potential impact of these factors are extensively examined, and as such, the manufacturer will develop an understanding of the influence of operating conditions upon process characteristics and the structural isoforms (Lee et al., 2012).

A comprehensive data package containing particulars of the manufacturing process, including the development of expression vectors and cell banks, cell culture or fermentation, harvest, purification, formulation, fill and finish, and storage information, must be compiled for the proposed biosimilar. In addition, details of dosage form and container closure system are investigated and documented (WHO, 2016). Demonstrating process understanding and control of the manufacturing process for the proposed biosimilar is a cornerstone for this package as it is for every biologic product.

## 5 HOW IS ANALYTICAL SIMILARITY ESTABLISHED?

### 5.1 Overall process, techniques, and methodology

Although there are no specific types of analyses or assays for evaluating all biologics, including potential biosimilars, the selection of analyses is influenced by the properties of the reference product. As such, similarity is determined on a case‐by‐case basis and the exact requirements can vary across regulatory agencies (Markenson, Alvarez, Jacobs, & Kirchhoff, 2017). The aim of an analytical similarity assessment is to investigate structural and functional elements such as primary structure, glycosylation, post‐translational modifications, purity, charge heterogeneity and higher order structure, as well as bioactivity features that may impact the clinical properties of the proposed biosimilar. For example, structural and functional characterization of a proposed biosimilar to Rituxan (rituximab) was conducted using multiple state‐of‐the‐art analytical tools (Visser et al., 2013) (Table 3).

**Table 3:** An overview of the characterization of mAb biosimilars (Visser et al., 2013)

Category	Quality attribute	Methods
Physicochemical characterization		
Primary structure	Amino acid sequence	Red. RP‐HPLC–ESI–MS peptide mapping, intact mass of whole mAb, HC and LC by RP‐HPLC–ESI–MS, Red. RP‐HPLC‐UV peptide mapping
Higher order structure	Disulfide bridging Free thiols Secondary and quaternary structure Thermodynamic stability	Non‐red. RP‐HPLC‐ESI–MS peptide mapping Ellman's assay CD, FTIR, HDX‐MS, X‐ray DSC
General charge heterogeneity and amino acid modifications	0 K variant, acidic variants, basic variants, Gln‐variant, Lys‐variant, amidated proline Glycation Oxidation/deamidation/C‐terminal variants	CEX digested/undigested Boronate affinity RP‐HPLC‐UV/MS peptide mapping
Glycosylation	Galactosylation, sialylation, mannosylation, afucosylation, bisecting GlcNAc, NGNA, α‐galactose, qualitative glycosylation pattern	NP‐HPLC‐FL
Size heterogeneity	Monomer, low molecular weight (LMW) and high molecular weight (HMW) variants (aggregates) Heavy chain (HC), light chain (LC), aglycosylated HC, clipped variants Monomer, LMW (e.g., half antibodies and HHL variant) and HMW variants Subvisible particles Visible particles	SEC, AF4 Red. CE‐SDS Non‐red. CE‐SDS Light obscuration (PhEur, ≥10 μm and >25 μm) Visual inspection (PhEur)
Functional characterization		
Target and receptor binding	FCRn binding SPR FcγR binding (FcγRIa, FcγRIIa, FcγRIIb, FcγRIIIa(F158), FcγRIIIa(V158), FcγRIIIb) SPR	
Bioactivity	CD20 target binding CDC potency ADCC potency Apoptosis	Cell‐based binding assay Cell‐based CDC assay Cell‐based ADCC assay Cell‐based apoptosis assay

ADCC, antibody‐dependent cell‐mediated cytotoxicity; AF4, asymmetric flow‐field fractionation; CD, circular dichroism; CDC, complement dependent cytotoxicity; CE‐SDS, capillary electrophoresis with sodium dodecyl sulfate; CEX, cation‐exchange chromatography; DSC, differential scanning calorimetry; ESI, electrospray ionization mass; FL, fluorescence; FTIR, fourier transform infrared; HDX, hydrogen deuterium exchange; HPLC, high‐performance liquid chromatography; LC, liquid chromatography; mAb, monoclonal antibody; MS, mass spectrometry; NP, normal phase; RP, reverse phase; SEC, size‐exclusion chromatography; SPR, surface plasmon resonance; UV, ultraviolet.

Reproduced from Visser et al. (2013).

A variety of techniques may be used to assess analytical similarity, and a few examples are outlined below. However, it is important to note that other techniques may be appropriate for the biosimilar in development, and future advances must also be considered.

The primary structure of the potential biosimilar and the reference product must be identical (FDA, 2015b); thus, it is important to use a multifaceted approach. Electrospray ionization–mass spectrometry (ESI–MS) may be used to confirm the primary structure and molecular mass of the potential biosimilar and the reference product for comparison at the intact molecular level. For mAbs, the potential biosimilar and the reference product are often evaluated at the domain level by enzymatic digestion into 25 KD subunits, followed by analysis using liquid chromatography–mass spectrometry (LC–MS) (Chevreux, Tilly, & Bihoreau, 2011) At the peptide level, the potential biosimilar and reference product are enzymatically digested into peptides and analyzed using LC–MS peptide mapping techniques. Furthermore, sequencing of the N‐terminal may be conducted using Edman degradation methods to evaluate the primary structure of the variable domains at the individual amino acid level (Edman, 1949; Visser et al., 2013). De novo sequencing of the entire amino acid sequence is typically accomplished using a combination of Edman degradation and LC–MS/MS sequencing techniques.

Major glycan structures that are attached to the protein can be identified using ESI–MS. A glycan profile is obtained by removal of glycans from the protein, followed by separation of the individual glycans using chromatographic techniques such as high‐performance anion‐exchange chromatography with pulsed amperometric detection (HPAEC‐PAD) or normal phase liquid chromatography. A common technique is enzymatic removal of N‐linked glycans using peptide N‐glycosidase F (PNGase F), followed by labeling the release N‐linked glycans with the fluorescent probe 2‐aminobenzamide, and separation by normal phase chromatography and fluorescence detection (Jung et al., 2014; Visser et al., 2013). Structures and glycosidic linkages are confirmed by sequential digestion using various exoglycosidases, followed by chromatographic analysis. Other techniques, such as sialic acid analysis with 1,2‐diamino‐4,5‐methylenedioxybenzene and monosaccharide analysis provide further details of specific glycan moieties (Jung et al., 2014; Visser et al., 2013).

Identification of the Fc glycosylation pattern is a key consideration during the development of mAb biosimilars, since the glycan chains in the Fc region can substantially alter protein activity and the PK profile, and in some cases, antigenicity. For example, glycosylation profiles, which contain appreciable levels of certain alpha‐gal structures, can confer the potential for undesired immunogenicity (Berger, Kaup, & Blanchard, 2012). Another example, improper or missing one or more glycosylation sites of rituximab may diminish the therapeutic effect of a biologic (Schiestl et al., 2011). Classes of glycan structures include O‐linked glycans bound to the protein through a serine or threonine linkage and N‐linked glycans, which bind to an asparagine that is part of the glycosylation consensus sequence primarily located on a conserved site in the CH2 domain. However, N‐linked glycans can be located in variable regions of a mAb, as seen with cetuximab (Qian et al., 2007). Typically, LC–MS subunit analysis is used to reveal the domain of the attached glycan structures and a peptide map is used to identify the location of the amino acid backbone with the glycan structure.

Post‐translational modifications often have a role in protein activity and can affect the function, stability, bioavailability, and immunogenicity of a protein. Disulfide bond pairings are evaluated using a native peptide map with MS detection, and the number of sulfhydryl groups per protein may be determined by Ellman's assay. Deamidation and oxidation sites are often evaluated using peptide mapping. Glycation and binding of glucose to lysine residues can be analyzed using boronate affinity chromatography. C‐terminal lysine can be evaluated by intact MS, peptide mapping, and ion exchange chromatography (IEC) in conjunction with carboxypeptidase B treatment, that cleaves the C‐terminal lysine, changing the charge profile (Jung et al., 2014; Visser et al., 2013) (Table 3).

The purity of the proposed biosimilar and the reference product is typically assessed using analytical techniques such as size‐exclusion (SE) high‐performance liquid chromatography (SE‐HPLC) and capillary gel electrophoresis (CGE). The quantity of aggregate and monomer in a protein are often evaluated using SE‐HPLC coupled with an ultraviolet (UV) spectrophotometer. Size‐exclusion chromatography (SEC) with multi‐angle light scattering and analytical ultracentrifugation sedimentation velocity (AUC‐SV) are characterization techniques used to support the SE‐HPLC analysis. SEC–multi‐angle light scattering is used to assess the weight‐average molar mass of the species eluting under each peak. AUC‐SV, an orthogonal technique to SE‐HPLC, is used to assess the level of aggregate species, and to detect large aggregates that cannot enter a chromatography column, and may otherwise be undetected. In addition, CGE is used to assess low molecular mass or fragment species.

Charge heterogeneity is determined using isoelectric focusing, IEC, and isoelectric capillary electrophoresis (iCE). These techniques are useful in separating protein products into acidic, main, and basic species. Isoelectric focusing is the classic gel technique that is stained for detection, IEC uses cation or anion exchange chromatography columns to separate intact proteins, and iCE uses a capillary to separate denatured proteins. IEC and iCE typically use a UV detector to detect eluting protein, which allows for integration and percentage area of each peak to be calculated (Jung et al., 2014). For mAb biosimilars, charge heterogeneity is assessed using IEC to separate intact proteins that are detected by UV (Visser et al., 2013). The use of a preparative IEC column allows for the isolation of purified peaks, followed by identification of the species eluting under each peak, and analysis of both reduced and non‐reduced sample preparations (Jung et al., 2014).

The higher order structure of the proposed biosimilar and the reference product is determined using X‐ray crystallography, which provides high resolution information on the protein fragments (Fab and Fc). Secondary structure is assessed using far‐UV circular dichroism and Fourier transform infrared spectroscopy, and the tertiary structure is evaluated using near‐UV circular dichroism and fluorescence spectroscopy. Thermal stability is determined using differential scanning calorimetry, which monitors the specific unfolding of the protein (Visser et al., 2013).

Additionally, techniques such as hydrogen deuterium exchange mass spectrometry (HDX‐MS) and proton nuclear magnetic resonance spectroscopy (^1^H‐NMR) can be used to characterize higher order structure (Wang and Li, 2014). HDX‐MS is used to evaluate localized small differences in structure, based on the observed ion exchange rates (Engen, 2009; Houde, Berkowitz, & Engen, 2011). ^1^H‐NMR is used to determine the environment of the protein protons as a comparative tool. However, the ability of these assessment techniques to identify meaningful differences remains undetermined.

In addition to structural characterization of biosimilars, key functional tests are required to assess biologic potency and activity, such as target and receptor binding, complement binding assays, cell‐mediated toxicity, and cytotoxicity, where appropriate. The functional evaluation of potential biosimilars is based on the mechanism(s) of action of the reference product as reported in the scientific literature (FDA, 2015b). Unlike the structural similarity assessment described above, which is accomplished using a common strategy for biosimilars, the functional similarity assessment is unique for each biosimilar.

The potency of the proposed biosimilar and the reference product is determined using cell‐based assays, which are used to examine the ability of the antigen to bind to its target and neutralize its biologic activity. Binding of the potential biosimilar to the target antigen is evaluated using target antigen binding assays, such as enzyme‐linked immunosorbent assay, surface plasmon resonance, or flow cytometry (Visser et al., 2013). These techniques are also used to assess the binding of the potential biosimilar to pertinent receptors. Table 4 provides an example of the functional assessments of a proposed biosimilar to Remicade (infliximab) and highlights the diverse nature of the functional assessment of a proposed biosimilar (Jung et al., 2014).

**Table 4:** Functional characterization of a proposed biosimilar to Remicade (Jung et al., 2014)

Category	Quality attribute	Techniques
Target and receptor binding	TNF binding	ELISA and cell‐based binding assay
	FcRN	SPR
	C1q	ELISA
Bioactivity	TNF neutralization	Cell‐based TNF neutralization assay
	Apoptosis	Cell‐based apoptosis assay
	CDC	Cell‐based CDC assay

CDC, complement dependent cytotoxicity; ELISA, enzyme‐linked immunosorbent assay; SPR, surface plasmon resonance; TNF, tumor necrosis factor.

### 5.2 US FDA guidance on the statistical aspects of establishing analytical similarity

A three‐tier approach to the statistical evaluation of analytical similarity between the proposed biosimilar and the reference product has been outlined by the FDA (Chow, Song, & Bai, 2016; FDA, 2015b). The CQAs are identified and divided into three tiers based on a risk assessment of their impact on biological activity, PK/PD, safety, and immunogenicity.

CQAs that have a high impact on biological activity, safety, or immunogenicity with results from the analytical testing amenable to statistical evaluation are assigned to a tier 1 analysis, which involves an equivalency analysis between the proposed biosimilar and the US reference product. CQAs assessed to have moderate impact on these risks and with results from the analytical analysis amenable to statistical analysis are assigned to a tier 2 analysis. This involves a quality range analysis between the proposed biosimilar and the US reference product. CQAs that have low or no impact on these risks or have results that are not amenable to statistical analysis, regardless of risk ranking, are assigned to a tier 3 analysis. This involves raw data or graphical presentation of results between the proposed biosimilar and the US reference product.

## 6 THE ROLE OF COMPARATIVE IN VIVO NONCLINICAL STUDIES

Nonclinical and clinical studies form the backbone of the efficacy and safety dossier of the reference product, and indeed for all new drug applications. In contrast, regulatory agencies recognize that comprehensive nonclinical evaluations are not required for the approval of biosimilars because the efficacy and safety of the reference product have previously been established, and analytical similarity to the biosimilar has been shown. Nonclinical in vivo studies are conducted after analytical characterization of the biosimilar, and are designed to address any specific residual analytical uncertainty and, in some jurisdictions, ensure safe use in humans.

Differences in cell lines, formulation, and processes between the proposed biosimilar and the reference product may warrant comparative nonclinical toxicity studies to ensure that human safety is not compromised (EMA, 2015; FDA, 2015b). Since the nonclinical toxicity profile of the reference product has already been established by the originator, unexpected toxicity findings with the biosimilar in such studies can be indicative of impurities or other factors that warrant further evaluation. Although global guidelines on biosimilar development are largely aligned in terms of the analytical and clinical aspects, there is substantial variability in the amount and type of in vivo nonclinical data required, with the EMA guidelines recommending minimal to no use of in vivo assays (EMA, 2015) whereas other countries, such as Japan and China, require more extensive toxicity studies (Health Canada, 2016; PMDA, 2009).

There are limitations to conducting traditional nonclinical studies in the context of establishing biosimilarity. These include ethical concerns regarding the use of nonhuman primates, together with the small sample size typically used in nonclinical studies of biologics. Hence, because of species differences and the small sample size, these studies are considered less informative than larger, statistically powered clinical trials (EMA, 2015; Van Meer et al., 2015). In addition, safety (including immunogenicity) in nonclinical species, even nonhuman primates, is not considered predictive of the potential for immunogenicity in humans (Ponce et al., 2009). In light of these limitations and concerns, fit‐for‐purpose comparative nonclinical in vivo evaluations may be needed to address specific residual analytical uncertainty or to meet regulatory requirements in some jurisdictions. In certain circumstances, such as identification of impurities or bridge manufacturing scale‐up, nonclinical studies of the biosimilar only (and not the reference product) may be appropriate.

## 7 ESTABLISHING CLINICAL EFFICACY AND SAFETY SIMILARITY

The clinical assessment of similarity of a proposed biosimilar to the reference product involves comparative PK/PD, immunogenicity, and efficacy and safety studies. Establishing PK and PD similarity is a key part of the development of biosimilars, as it is not possible to accurately determine the PK and PD profiles based solely on nonclinical studies (FDA, 2015b). Furthermore, data from analytical and comparative PK studies can be used as a “bridge” that permits use of a single (US or EU) reference product in larger, comparative clinical efficacy studies.

Comparative clinical efficacy and safety trials are conducted, including immunogenicity assessments and, in some cases, confirmatory PK/PD studies to demonstrate clinical similarity of the proposed biosimilar to the reference product. The aim of clinical comparative studies is not to re‐establish efficacy and safety, but to identify any clinically meaningful differences between the proposed biosimilar and the reference product, and to resolve residual uncertainty.

## 8 THE ROLE OF COMPARATIVE IMMUNOGENICITY ASSESSMENT

Treatment with biologic products, including biosimilars, may provoke an immunogenic response, which could potentially alter the PK, efficacy, and safety properties of these agents (Bendtzen, 2012; Pendley, Schantz, & Wagner, 2003). Several factors have the potential to trigger an immunogenic response, including structural differences, aggregates, heterologous protein/amino acid mismatch, host cell proteins or other impurities, and varying glycosylation patterns (Liu, Zou, Sadhu, Shen, & Nock, 2015). Furthermore, concomitantly administered immunosuppressive therapy and chemotherapy can influence immunogenicity (Buttel et al., 2011). Therefore, careful examination of the formation of anti‐drug antibodies in patients treated with biologics is critical throughout development and during post‐marketing surveillance of biosimilars.

## 9 THE SCIENTIFIC PRINCIPLES OF EXTRAPOLATION ACROSS INDICATIONS

Extrapolation is a scientific and regulatory term that describes the approval of a biosimilar for use in an indication held by the reference product, which is not directly studied in a comparative clinical trial with a biosimilar. Extrapolation is based on establishing a similar mechanism of action for the biosimilar in various disease indications (FDA, 2015b). Extrapolation of clinical data can reduce or eliminate the need for studies in multiple indications, and therefore, may increase access to biosimilars sooner. Although the decision to extrapolate data from one indication to another is made on a case‐by‐case basis, with strong scientific justification, based on the totality of evidence, the concepts are supported by the EMA and the US FDA regulatory guidelines (EMA, 2015; FDA, 2015b).

## 10 CONCLUSIONS

Despite many challenges, the development of biosimilars continues in earnest. Biosimilarity must be established based on the totality of evidence, from structural and functional assessment through nonclinical and clinical studies, adopting a tailored approach throughout development. It is clear that we must think differently when developing biosimilars, especially when defining CQAs and setting endpoints for nonclinical and clinical studies. The arrival of biosimilars challenges the healthcare community to learn and understand the scientific basis of similarity to the reference product using a stepwise approach. An increased awareness is needed to understand that clinical studies are a blunt instrument in the development of biosimilars, and that analytical evaluation is a far more sensitive tool in assessing similarity.
